# A machine learning framework for predictive interpretation of variants of uncertain significance in hereditary cancer

**DOI:** 10.3389/fsysb.2026.1828263

**Published:** 2026-08-04

**Authors:** Nayeema Nizamuddin, Soham Biswas, Akshaykumar Zawar, Poonam Deshpande, Layal Yasin, Prashanth N. Suravajhala

**Affiliations:** 1 Bioclues.org, Hayatnagar, Hyderabad, India; 2 Department of Biosciences, Systems Genomics Lab, Manipal University Jaipur, Jaipur, India; 3 GeneSpectrum Life Sciences LLP, Pune, Maharashtra, India; 4 Department of Life Sciences, School of Science and Mathematics, DES Pune University, Pune, Maharashtra, India; 5 Department of Pediatric Oncology, Haematology and Clinical Immunology, Medical Faculty, Heinrich-Heine-University, Düsseldorf, Germany

**Keywords:** clinvar, hereditary cancer, machine learning, SHAP, variant prioritization, variants of uncertain significance

## Abstract

**Introduction:**

Variant interpretation remains a major bottleneck in clinical genomics, with variants of uncertain significance (VUS) representing a critical unresolved challenge due to insufficient evidence for definitive classification. Existing in silico tools exhibit variable and often inconsistent performance complicating clinical decision-making, particularly in the context of hereditary cancer genomics.

**Methods:**

In this study, we developed a machine learning framework trained on 1,04,646 high-confidence ClinVar germline variants (3-star+ review status) annotated with Ensembl VEP (v114, GRCh38) and CADD v1.6 pathogenicity scores to classify variants as Pathogenic or Benign, subsequently applying the trained model to reclassify 40894 ClinVar VUS. Train/test partitioning was performed at the variant level (80/20 split) to prevent data leakage, with hyperparameter optimization via GridSearchCV and performance assessed by 10-fold cross-validation. Four classifiers were evaluated viz. Logistic Regression, Support Vector Machine, Random Forest and XGBoost, with Random Forest achieving the highest performance (AUC-ROC = 0.9995, 95% CI: 0.9993–0.9997; 10-fold CV AUC = 0.9992 ± 0.0004). Probability thresholds of P ≥ 0.80 (Pathogenic) and P <= 0.20 (Benign) were derived from Precision-Recall curve analysis, achieving empirically validated precision of 99.63% and 99.77% respectively on held-out test variants.

**Results and Discussion:**

Applied to 40,894 ClinVar VUS, the model reclassified 19393 (47.4%) as Likely Pathogenic and 8,957 (21.9%) as Likely Benign, while 12,544 (30.7%) were conservatively retained as uncertain. External validation on 7,462 ENIGMA-classified BRCA1/BRCA2 variants from the BRCA Exchange database, completely independent of the ClinVar training data demonstrated an overall concordance of 98.83% (AUC = 1.0000). Further validation of VUS reclassification against 671 variants classified as VUS in ClinVar but definitively classified by ENIGMA yielded an overall concordance of 89.57% (Pathogenic: 96.4%, Benign: 87.4%). SHAP-based explainability analysis confirmed that predictions were predominantly driven by biologically interpretable features, including CADD Phred score, VEP functional impact tier, variant consequence class and population allele frequency, consistent with ACMG/AMP evidence criteria. This reproducible pipeline provides a clinically grounded computational approach to VUS triaging in precision oncology, with external validation supporting its generalizability to independent hereditary cancer gene datasets.

## 1 Introduction

The rapid adoption of next-generation sequencing (NGS) technologies has revolutionized the identification of germline variants associated with hereditary cancer syndromes. While pathogenic and benign variants can often be classified with high confidence using established clinical guidelines, a substantial proportion remain as variants of uncertain significance (VUS; Price et al., 2018). These VUS pose a major challenge in clinical genetics as their interpretation directly affects patient counseling, risk assessment and medical management (Fowler and Rehm, 2024; Zawar et al., 2025; McLaren et al., 2016). The American College of Medical Genetics and Genomics (ACMG), Association of Molecular Pathology (AMP) and The Association for Clinical Genomics Sciences (ACGS) provide widely accepted criteria for variant classification, incorporating population frequency, computational predictions, functional studies, segregation data and clinical observations (Richards et al., 2015). However, these criteria often yield inconclusive results for rare or novel variants, particularly in large gene panels or exome sequencing datasets. Consequently, computational approaches leveraging high-dimensional genomic and functional annotations have emerged as essential tools to aid in the interpretation of VUS. For instance, the real-time novel variant classification (RENOVO) algorithm employs deep neural networks to predict the pathogenicity of missense variants, demonstrating high accuracy and potential clinical utility (Favalli et al., 2021). Similarly, MARGINAL, a machine learning (ML) model developed for BRCA1/2 variants from ClinVar, achieved 98.1% accuracy and an F1 score of 0.975, outperforming existing methods and illustrating ML’s potential in personalized cancer risk assessment (Karalidou et al., 2022).

The ML models have also been applied to predict pathogenicity using genetic and clinical data. For example, DeepGestalt trained on 150,000 United Kingdom Biobank participants, accurately predicted type-2 diabetes risk (AUC-ROC = 0.88), highlighting ML’s utility in personalized medicine (Lugner et al., 2024). In germline variant curation, an ML model trained on 2752 BRCA1/2 variants and validated on 200 achieved 96.5% accuracy with high sensitivity and specificity, improving efficiency and consistency in clinical variant classification (Ravichandran et al., 2019). Deep learning has also been used in cancer genomics to classify cancer types and identify driver genes using convolutional neural networks on TCGA data, achieving 92.6% accuracy (Zeng et al., 2021). Integrative approaches combining ACMG/AMP guidelines with variant annotations via penalized logistic regression have been shown to generate probabilistic pathogenicity scores resolving more VUS than guideline-only methods (Nicora et al., 2022). On the other hand, platforms like CancerVar further demonstrate the power of AI-driven interpretation, leveraging deep learning frameworks aligned with AMP/ASCO/CAP 2017 guidelines to predict oncogenicity for millions of somatic mutations, thereby automating interpretation and supporting clinical decision-making (Li et al., 2022). The Ensembl Variant Effect Predictor (VEP) has become a cornerstone tool in variant annotation and prioritization, predicting molecular consequences, phenotype associations, allele frequencies and deleteriousness scores while providing flexible interfaces for large-scale analyses (Hunt et al., 2022).

Recent studies have demonstrated the potential of ML to integrate diverse variant-level features such as allele frequency, molecular consequence, protein impact predictions, conservation scores and variant type to prioritize VUS for further investigation. Semi-quantitative refinements of ACMG/AMP guidelines, such as the Sherloc framework, further improve variant interpretation by assigning weighted evidence scores and probabilistic stratification, enabling more nuanced resolution of VUS while maintaining clinical conservatism (Nykamp et al., 2017). In contrast, traditional tools that rely on single features or neglect transcript-level context may miss clinically relevant variant effects. Complementary to this, ML-based pathogenicity stratification models trained on large curated resources such as ClinVar generate calibrated pathogenicity probabilities enabling systematic prioritization and redistribution of VUS reclassification (Kobayashi et al., 2024). Despite these advances, several methodological gaps persist. First, many existing ML approaches treat VUS as a distinct training class, even as VUS labels reflect evidential uncertainty rather than a true biological category, introducing circular reasoning into model training (Forrest et al., 2025). Second, transcript-level data leakage from inadequate train/test splitting strategies has not been systematically addressed (Sha et al., 2024). Third, classification thresholds are frequently set arbitrarily at 0.5 rather than anchored to established clinical evidence standards (Liu Y et al., 2022). In this work, we address these gaps by developing a binary ML classifier trained exclusively on variants with established clinical significance from ClinVar, with train/test partitioning performed at the variant level to prevent transcript-level leakage. Four classifiers, *viz.* Logistic Regression, Support Vector Machine, Random Forest and XGBoost were compared with probability thresholds grounded in the Bayesian ACMG/AMP framework (Tavtigian et al., 2020). The framework was externally validated on 7462 ENIGMA classified BRCA1/2 variants from the BRCA Exchange database (https://brcaexchange.org/release/76 last accessed on 15 May 2026), achieving 98.83% concordance and applied to 40,894 ClinVar VUS to generate computational reclassification predictions across a broad spectrum of hereditary cancer genes. Our findings demonstrate a scalable framework for integrating predictive modeling into variant interpretation pipelines and highlight its ability to support more efficient triaging of variants in clinical genomics practice.

## 2 Materials and methods

### 2.1 Data curation

Germline cancer variants were retrieved from ClinVar (last accessed May 2026), a publicly available repository of clinically annotated human genomic variants maintained by the National Center for Biotechnology Information (NCBI). The GRCh38-mapped VCF files were downloaded from the NCBI FTP server (https://ftp.ncbi.nih.gov/snp/latest_release/VCF/GCF_000001405.40.gz). To ensure only high-confidence variants, ClinVar review status, multiple submitters, no conflicts, encompassing classifications by expert panels or based on established practice guidelines were included. This stringency filter yielded 104,646 high-confidence germline variants comprising 40,175 Benign/Likely Benign, 23,578 Pathogenic/Likely Pathogenic and 40,894 VUS, reflecting a pathogenic to benign ratio of 1:1.7 consistent with the known distribution of curated variants in ClinVar. Unique variant identifiers were extracted from the filtered dataset and used to retrieve corresponding genomic records from the ClinVar GRCh38 VCF using BCFtools (version 1.21; Danecek et al., 2021), producing a subset VCF containing only variants of interest with precise chromosomal coordinates on the GRCh38 human reference assembly. We further attempted comparing our annotations with AION/Nostos Genomics (https://app.nostos-genomics.com/, last accessed on 30 April 2026) particularly on variants categorized by ClinVar and checked whether or not the classification is in agreement with each others’ annotation.

### 2.2 Variant annotation using VEP and pathogenicity scoring

Functional annotation was performed using the Ensembl Variant Effect Predictor (VEP, cache version 114, GRCh38; McLaren et al., 2016) against the GRCh38 reference assembly. VEP was configured to retrieve all available annotation fields in a single pass, including molecular consequence terms following sequence ontology nomenclature, functional impact tier classifications ranging from high to modifier, missense deleteriousness predictions from SIFT (Ng and Sift, 2003) and PolyPhen-2 (Adzhubei et al., 2013). The population allele frequencies from the Genome Aggregation Database (gnomAD; Gudmundsson et al., 2022; Karczewski et al., 2020) exome and genome datasets were considered across global ancestry groups along with gene symbols, transcript identifiers, MANE Select and MANE Plus Clinical transcript designations, principal isoform annotations, protein consequence and amino acid change information, and variant class assignments. Since each variant overlaps multiple transcripts, only the single most biologically relevant and representative transcript per gene was retained per variant, as designated by Ensembl. Parallel processing across 12 CPU threads was employed to handle the large variant volume efficiently.

**FIGURE 1:**
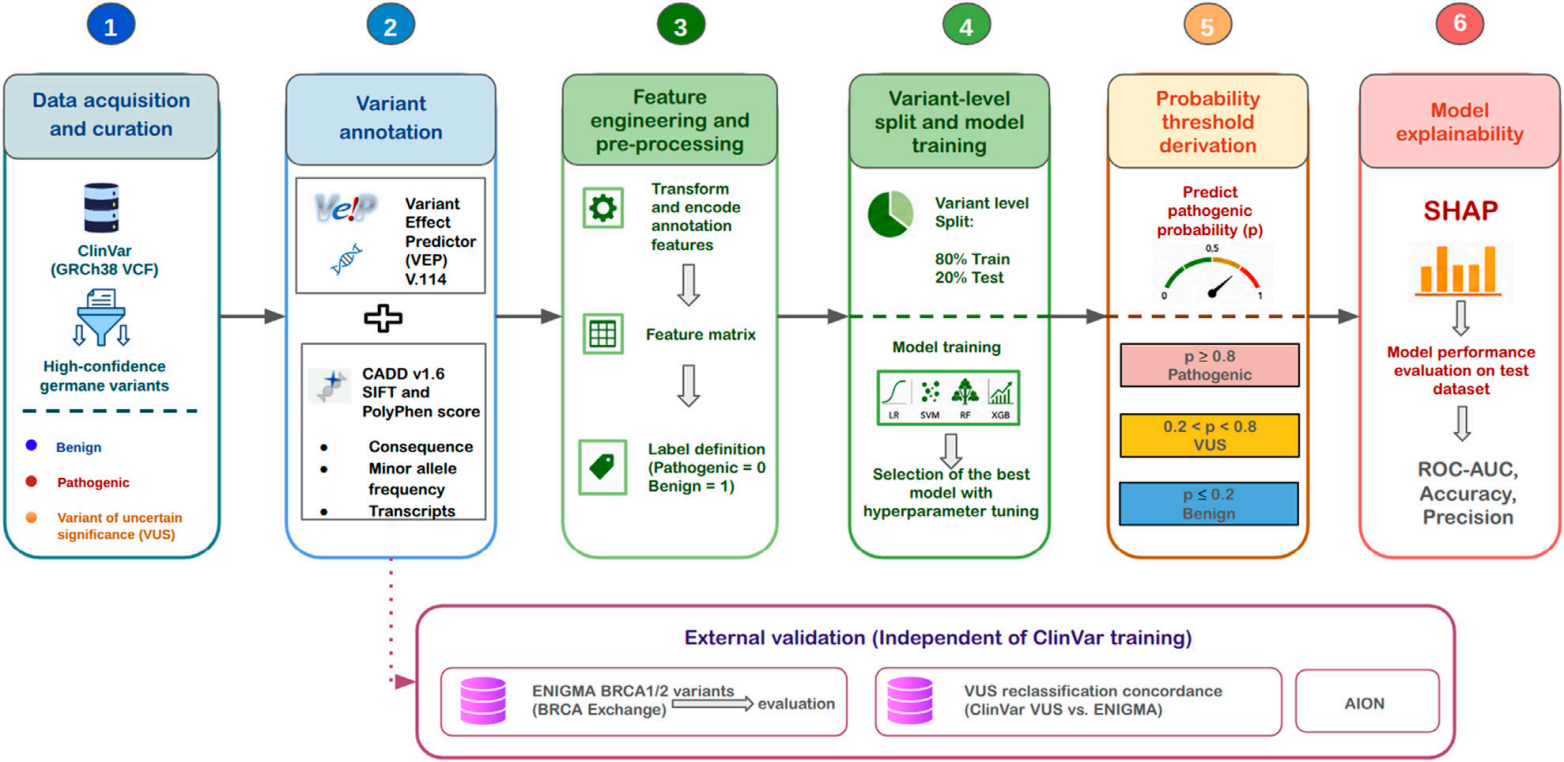
An overview of the machine learning framework for predictive interpretation of variants of uncertain significance (VUS) in germline cancer variants.

Additionally, genome-wide pathogenicity scores were obtained for all variants using the Combined Annotation Dependent Depletion (CADD) framework (https://cadd.bihealth.org/score last accessed on 30 April 2026, version 1.6, GRCh38; Rentzsch et al., 2019), which integrates diverse genomic annotations into a single PHRED-scaled deleteriousness score. The highest CADD PHRED score per variant was retained and merged with VEP annotations on a variant key comprising chromosome, position, reference and alternate allele, producing a final dataset of 104,639 variants each represented by a single annotation record combining functional and deleteriousness information. A schematic overview of the complete computational pipeline from data curation and VEP annotation through feature engineering, model training and application to ClinVar VUS and ENIGMA variants is shown in Figure 1.

### 2.3 Exploratory data analysis (EDA) and data pre-processing

Exploratory data analysis was performed on the annotated variant dataset to characterize class-specific distributions of key annotation features prior to model training. Distribution analyses were conducted for population allele frequencies, VEP functional impact categories, variant consequences, SIFT and PolyPhen predictions, CADD Phred scores, variant classes and biotype assignments across Pathogenic/Likely Pathogenic and Benign/Likely Benign classes. Pairwise correlations among continuous features were examined to identify redundant features, which were subsequently removed for training the model. Redundant population allele frequency columns were consolidated into a single maximum observed population allele frequency across all populations. The labeled dataset comprising Pathogenic/Likely Pathogenic and Benign/Likely Benign variants was used for training the model, while VUS were retained as a separate inference set for downstream reclassification. The labeled dataset, however, exhibited mild class imbalance, inversely proportional to class frequencies.

### 2.4 Feature engineering and label definition

Raw annotation fields from VEP and CADD were transformed into a structured numerical feature matrix suitable for machine learning. SIFT and PolyPhen-2 predictions were parsed to extract both the categorical label (e.g., deleterious, tolerated, probably_damaging, benign) and the associated numeric score where available. For other variants where these scores are undefined by design, the categorical label was assigned a dedicated missing category and the numeric score was encoded with a value of −1 to distinguish absence of evidence from a neutral prediction. Population allele frequency was represented by the maximum observed allele frequency across all global populations sourced from the gnomAD, with missing values considered as zero to reflect absence from population databases. While variant consequence terms were grouped from 69 unique VEP consequence terms into 11 biologically meaningful categories (missense, synonymous, frameshift, stop variant, canonical splice, splice region, inframe indel, UTR, intronic, regulatory and other) to reduce dimensionality, biological interpretable values were preserved. Biotype was simplified to a binary representation distinguishing protein-coding transcripts from all other transcript types. All categorical features were label-encoded using fixed mappings consistent across training, test and inference sets. Features exhibiting high pairwise correlation were excluded prior to finalization, including CADD RawScore (r = 0.97 with PHRED) and individual gnomAD exome and genome allele frequencies (r > 0.82 with maximum population allele frequency). Germline classification labels were derived from the ClinVar Germline classification field. Variants annotated as Pathogenic or Likely Pathogenic were assigned a positive binary label, and those annotated as Benign or Likely Benign were assigned a negative binary label, consolidating related sub-classifications into their respective categories. The VUS, however, were excluded from the initial training model and later retained exclusively as an inference set for downstream reclassification.

### 2.5 Train/test splitting strategy

A critical methodological consideration in variant classification is ensuring that the same variant does not appear in both training and test partitions, which would constitute data leakage and artificially inflate performance metrics. In our pipeline, since only the canonical transcript annotation was retained per variant, the dataset was already structured at the variant level with a perfect match between variants and annotation records. Train/test partitioning was therefore performed directly at the variant level using an 80/20 split ratio with stratification by class label to preserve the Pathogenic to Benign ratio across both partitions and a fixed random seed for reproducibility. This variant-level splitting strategy ensures that no variant contributes to both training and evaluation, providing an unbiased estimate of model performance on VUS. The training partition comprised 50,996 variants and the test partition comprised 12,749 variants. The VUS were withheld entirely from both partitions and used exclusively as an independent inference set for downstream reclassification.

### 2.6 Model training and comparison

Four supervised binary classifiers were trained on the labeled training partition to discriminate Pathogenic from Benign variants: Logistic Regression (LR; Sando Sperandei, 2014), Support Vector Machine with RBF kernel (SVM; Evgeniou and Pontil, 2001), Random Forest (RF; Rigatti SJ, 2017) and XGBoost gradient boosting (XGB; Chen and Guestrin, 2016). All implementations used the scikit-learn Python library (Pedregosa et al., 2011) except XGBoost which used the dedicated XGBoost Python library. The training set exhibited a mild class imbalance with a Pathogenic to Benign ratio of 1:1.7. To prevent bias toward the majority class during training, all classifiers employed class-weighted loss functions, assigning weights inversely proportional to class frequencies.

Systematic hyperparameter optimization was performed for Random Forest and XGBoost using grid search with five-fold cross-validation across a predefined parameter search space (Table 1). Model performance was evaluated on the held-out test partition using area under the receiver operating characteristic curve (AUC-ROC), along with precision and recall (P/R), F1 score and accuracies were tabulated. To obtain robust and unbiased performance estimates, ten-fold cross-validation was additionally performed on the training partition for the two best-performing models, albeit there was no significant difference in accuracy. The final model was selected based on the highest test set AUC-ROC, interpretability and computational efficiency.

**TABLE 1:** Hyperparameter optimization of classifiers employing 5-fold CV.

Classifier	Initial hyperparameters	Class imbalance	Hyperparameter optimization (GridSearchCV)	Best 5-fold CV AUC
Logistic regression	max_iter = 1,000	class_weight = balanced	-	-
SVM	kernel = rbf, probability = True	class_weight = balanced	-	-
Random forest	n_estimators = 200, max_depth = 20	class_weight = balanced	n_estimators = 300, max_depth = 10, min_samples_leaf = 2	0.9992
XGBoost	n_estimators = 200, max_depth = 6, learning_rate = 0.1	scale_pos_weight = 1.70	n_estimators = 100, max_depth = 4, learning_rate = 0.1, subsample = 0.8	0.9991

**TABLE 2:** Performance comparison of all four classifiers on the held-out test set (12,749 variants) prior to hyperparameter optimization. P = Pathogenic, B = Benign.

Classifier	ROC-AUC	Precision(P)	Recall(P)	F1(P)	Precision(B)	Recall(B)	F1(B)
Random forest	0.9991	0.99	0.99	0.99	0.99	0.99	0.99
XGBoost	0.9991	0.99	0.99	0.99	0.99	0.99	0.99
Logistic regression	0.9972	0.96	0.99	0.97	0.99	0.98	0.98
SVM	0.9957	0.95	0.99	0.97	0.99	0.97	0.98

**TABLE 3:** Performance of Random Forest and XGBoost following hyperparameter optimization.

Classifier	Test ROC-AUC	95% CI	10 fold CV AUC	CV std
Random forest	0.9995	0.9993–0.9997	0.9992	±0.0004
XGBoost	0.9995	0.9992–0.9997	0.9992	±0.0004

### 2.7 Probability threshold derivation

To translate continuous predicted probabilities into clinically interpretable classifications, a three-zone threshold strategy was adopted. Probability thresholds were first derived empirically from the Precision-Recall curve computed on the held-out test partition, identifying the lowest probability value at which the classifier achieved a minimum precision of 99% for the Pathogenic class at 0.52. To ensure a higher degree of clinical confidence beyond this data-driven minimum, more stringent thresholds of ≥0.80 and ≤0.20 were adopted for Pathogenic and Benign classifications respectively. These thresholds align closely with the Bayesian extension of ACMG/AMP guidelines which defines posterior probabilities of pathogenicity ≥ 0.90 and ≤ 0.10 as the quantitative boundaries for Pathogenic and Benign classification, respectively. The thresholds of ≥0.80 and ≤0.20 represent a slightly more inclusive adaptation that increases clinical utility by reducing the uncertain zone. The resulting three-zone classification scheme assigns variants with predicted probability ≥0.80 as Likely Pathogenic/Pathogenic, those ≤ 0.20 as Benign/Likely Benign, and those falling between the two thresholds as VUS, reflecting genuine model uncertainty rather than a forced binary decision. The sensitivity of classification outcomes to threshold selection was assessed by evaluating variant distributions across a range of Pathogenic thresholds (0.70, 0.80, 0.90, 0.95).

### 2.8 Model explainability

To assess the biological interpretability of the best-performing model, SHapley Additive exPlanations (SHAP; Lundberg and Lee, 2017) values were computed using the TreeExplainer algorithm, which provides exact Shapley value decompositions for tree-based ensemble models. SHAP values were computed on a random sample of 1,000 test variants to balance computational efficiency. Global feature importance was quantified as the mean absolute SHAP value per feature across all sampled variants, providing a model-agnostic ranking of individual feature contributions to predicted pathogenicity. The directionality of feature effects was verified against biologically expected relationships to confirm that model decisions are driven by interpretable genomic signals consistent with established ACMG/AMP evidence criteria rather than spurious correlations.

### 2.9 External validation

To assess the generalizability of the trained model to independent data beyond the ClinVar training set, external validation was performed using expert-curated variant classifications from the Evidence-based Network for the Interpretation of Germline Mutant Alleles (ENIGMA) consortium, accessed through the BRCA Exchange database (Cline et al., 2018). ENIGMA provides definitive classifications for BRCA1 and BRCA2 variants based on multifactorial likelihood models incorporating functional assay evidence, familial segregation data, co-occurrence analysis and population frequency data representing a classification standard completely independent of ClinVar submissions.

ENIGMA-classified variants were annotated using VEP and CADD with identical parameters and software versions as the training pipeline. The same feature engineering transformations and label encodings derived from the training partition were applied to ensure consistency. Model predictions were generated using the trained Random Forest classifier and compared against ENIGMA classifications to assess concordance. External validation was conducted at two levels. First, overall model performance was assessed on all ENIGMA-classified Pathogenic and Benign BRCA1/BRCA2 variants to evaluate the generalizability of the trained classifier to an independent hereditary cancer gene dataset. Second, to specifically validate VUS reclassification performance, variants classified as VUS in ClinVar but with definitive ENIGMA classifications were identified and the concordance between model predictions and ENIGMA classifications was assessed.

## 3 Results and discussion

### 3.1 Data characteristics and exploratory analysis

**FIGURE 2:**
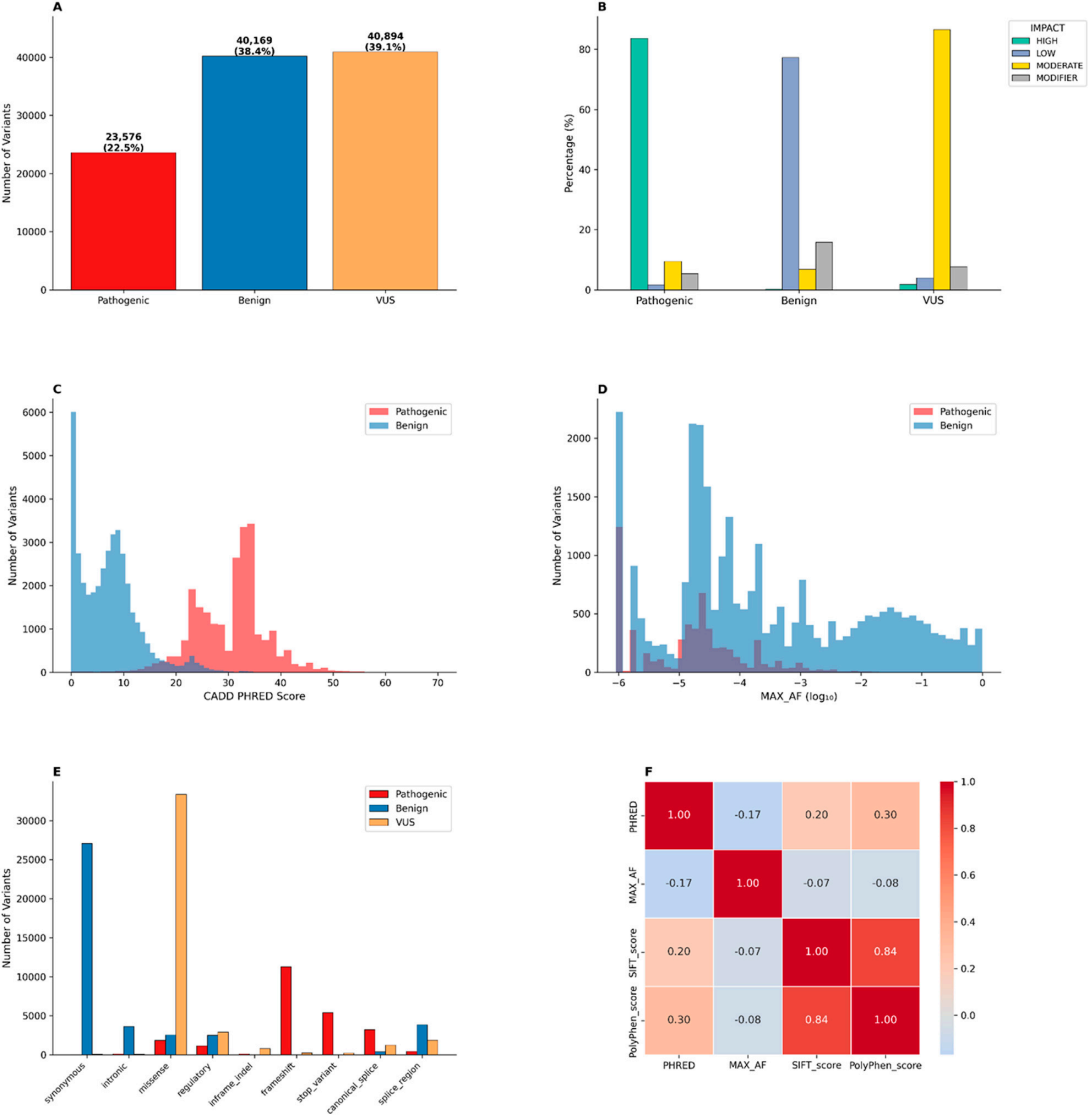
Exploratory data analysis of the ClinVar variant dataset. **(A)** Class distribution of the full dataset across Pathogenic/likely pathogenic, benign/likely benign and Variant of Uncertain Significance categories. **(B)** Functional impact category distribution across the three variant classes. **(C)** CADD PHRED score distributions for pathogenic and benign variants. **(D)** Population allele frequency distributions for Pathogenic and Benign variants. **(E)** Variant consequence type distribution across the three classes. **(F)** Pairwise Pearson correlation matrix of continuous annotation features. Pathogenic: Red; Benign: Blue; VUS: Orange. **(A)** Class distribution **(B)** IMPACT distribution by class **(C)** CASS PHRED score distribution **(D)** Population allele frequency distribution **(E)** Consequence distribution by class **(F)** Feature correlation matrix.

Following retrieval from ClinVar, VEP annotation and CADD scoring, the final dataset consisted of 104,646 variants. The labeled variant set comprised 23,578 Pathogenic/Likely Pathogenic and 40,175 Benign/Likely Benign variants, reflecting a pathogenic to benign ratio of 1:1.7. A total of 40,894 VUS were retained exclusively as the inference set and withheld from all model training and evaluation steps. Exploratory analysis of the labeled variant set revealed strong class-discriminative signals across multiple annotation tiers (Figure 2). Pathogenic variants were substantially enriched for High functional impact consequences (83.7%) compared to benign variants (0.2%) and were associated with frameshift, stop-gain and canonical splice variant consequence types. In contrast, Benign variants were associated with Low functional impact (77.3%) driven largely by synonymous variants, while VUS were overtly classified as Moderate impact (86.6%), corresponding to missense variants reflecting the inherent ambiguity of this consequence class in clinical interpretation.

CADD Phred scores showed strong class separation, with Pathogenic variants exhibiting substantially higher mean deleteriousness scores compared to Benign variants (mean PHRED 30.57 vs. 7.27 respectively) confirming the discriminative value of genome-wide deleteriousness scoring. Population allele frequency analysis revealed that Pathogenic variants were significantly rarer in global populations compared to Benign variants (mean MAX_AF 0.000083 vs. 0.030 respectively) with 75% of pathogenic variants entirely absent from gnomAD. SIFT and PolyPhen-2 scores were available for missense variants only, representing 62% and 65% missing values respectively across the full dataset, reflecting their design restriction to amino acid substitutions. Among scored variants, pathogenic variants showed higher predicted deleteriousness by both tools compared to benign variants confirming their class-discriminative value where available. Pairwise correlation analysis of continuous features identified high redundancy between CADD RawScore and PHRED (r = 0.97) and between individual gnomAD exome and genome allele frequencies and the maximum population allele frequency (r > 0.82), supporting their exclusion from the final feature matrix. The final feature set consisted of nine variables retained for model training.

### 3.2 Model comparison

**FIGURE 3:**
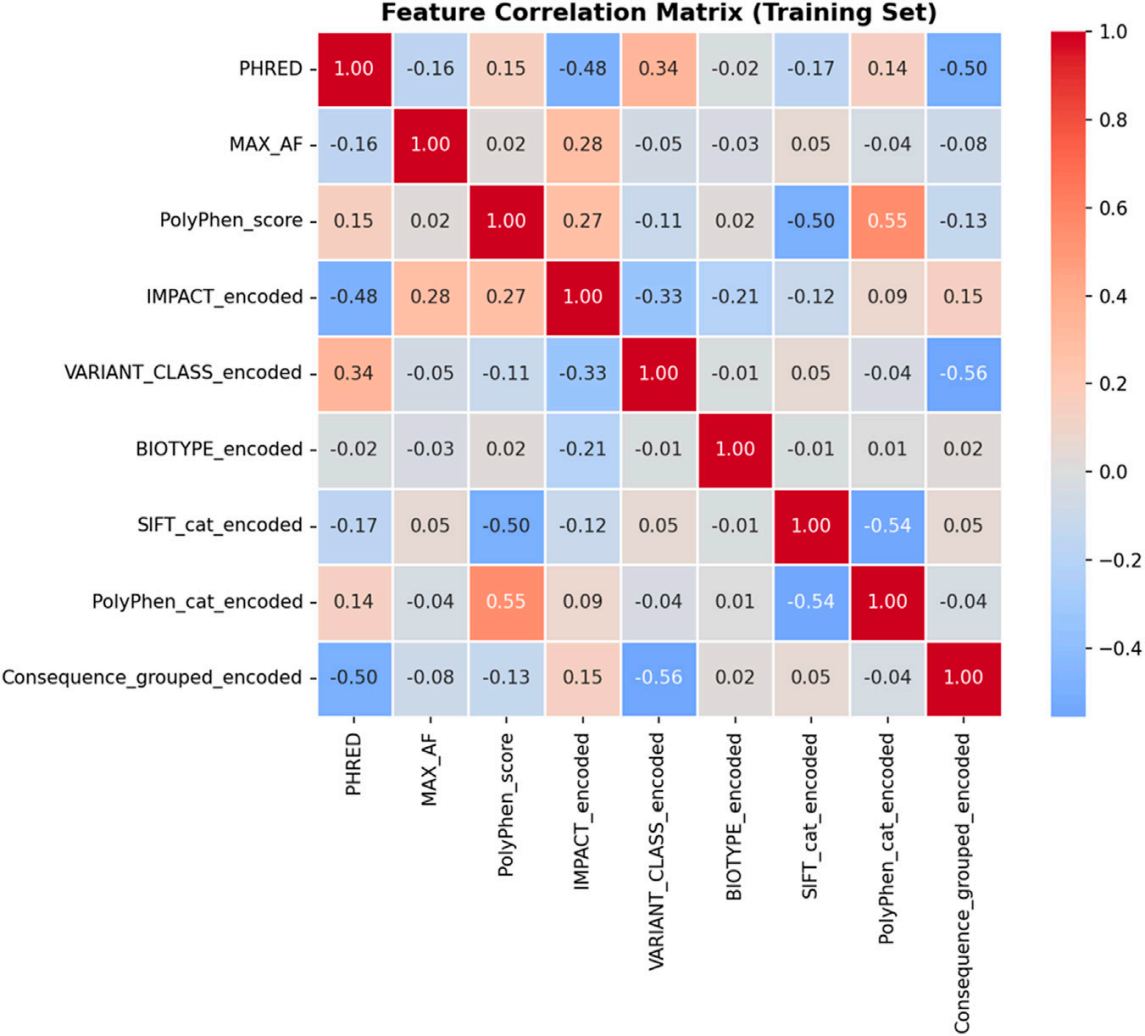
Pairwise Pearson correlation matrix of the nine candidate features used for model training. Highly correlated features were excluded prior to model training.

Following variant-level train/test partitioning, all four classifiers were trained on 50,996 variants and evaluated on a held-out test set of 12,749 variants with zero variant overlap confirmed. Prior to model training, pairwise correlation analysis of the nine candidate features was performed to identify redundant variables. CADD RawScore was found to be highly correlated with CADD Phred (r = 0.97) and was excluded and SIFT numeric score was found to be highly correlated with PolyPhen numeric score (r = 0.85) yielding a final feature matrix of nine variables (Figure 3). All four classifiers were first evaluated at their initial configurations prior to hyperparameter optimization (Table 1).

**FIGURE 4:**
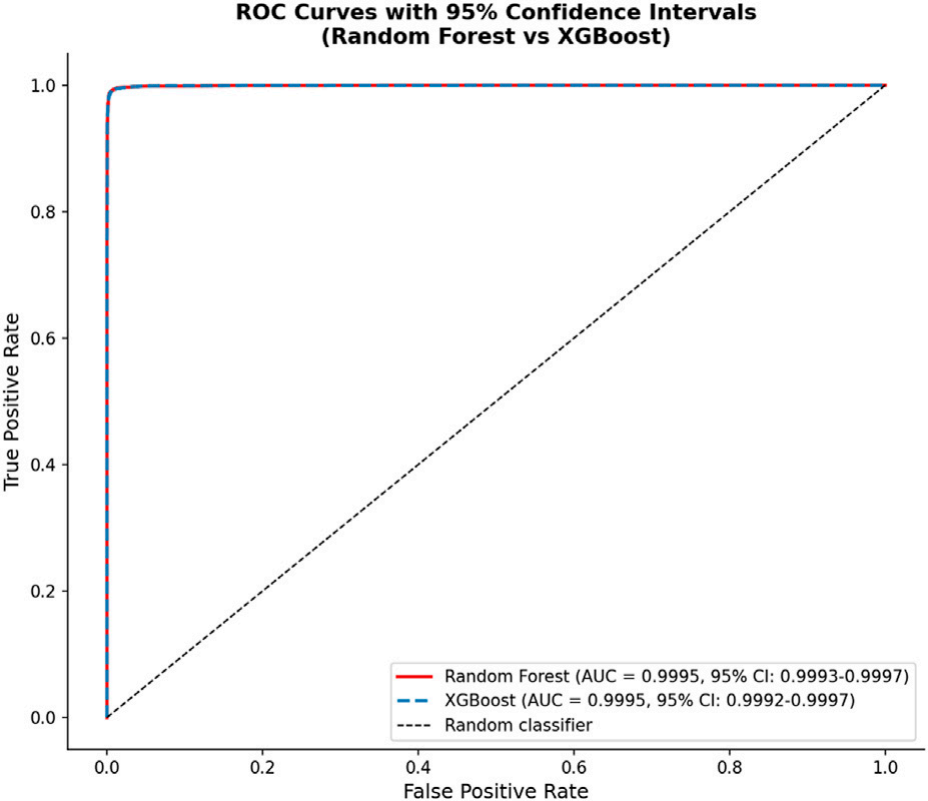
ROC curves with 95% confidence intervals for tuned Random Forest and XGBoost classifiers on the held-out test set (n = 12,749 variants).

ROC-AUC ranged narrowly from 0.9957 (SVM) to 0.9991 (Random Forest and XGBoost), consistent with the observation that discriminative signal is primarily attributable to the feature representation rather than classifier-specific learning capacity. The narrow performance range across diverse learning algorithms further confirms the robustness of the selected feature set. Random Forest and XGBoost achieved the highest and equivalent ROC-AUC of 0.9991 and were therefore selected for systematic hyperparameter optimization using grid search with five-fold cross-validation (Table 2). Following optimization, both models achieved a test set ROC-AUC of 0.9995 with 95% confidence intervals of 0.9993–0.9997 and 0.9992–0.9997 respectively, estimated by bootstrap resampling over 1,000 iterations (Figure 4). Ten-fold cross-validation on the training partition confirmed stable and generalizable performance for both models, with a mean ROC-AUC of 0.9992 ± 0.0004 indicating absence of overfitting (Table 3).

Random Forest was selected as the final model for all downstream analyses based on equivalent performance to XGBoost, superior computational efficiency and greater compatibility with SHAP-based explainability frameworks for tree ensemble models. SVM achieved the lowest ROC-AUC (0.9957) with substantially longer training time offering no practical advantage for this application.

### 3.3 Probability threshold derivation and three-zone classification

To translate continuous predicted probabilities into clinically interpretable classifications, probability thresholds were derived empirically from the Precision-Recall curve computed on the held-out test set of 12,749 variants for both Random Forest and XGBoost (Supplementary Figure S1). Both models achieved identical Average Precision of 0.9993, with curves overlapping throughout further confirming comparable performance. The data-driven analysis identified a minimum Pathogenic threshold of 0.52, at which the classifier achieved a Pathogenic precision of 99% with a corresponding recall of 99.1%, and a Benign threshold of 0.30. To ensure a higher degree of clinical confidence beyond these data-driven minima, more stringent thresholds of ≥0.80 and ≤0.20 were adopted for pathogenic and benign classifications respectively, consistent with probabilistic frameworks employed in comparable variant classification studies.

**FIGURE 5:**
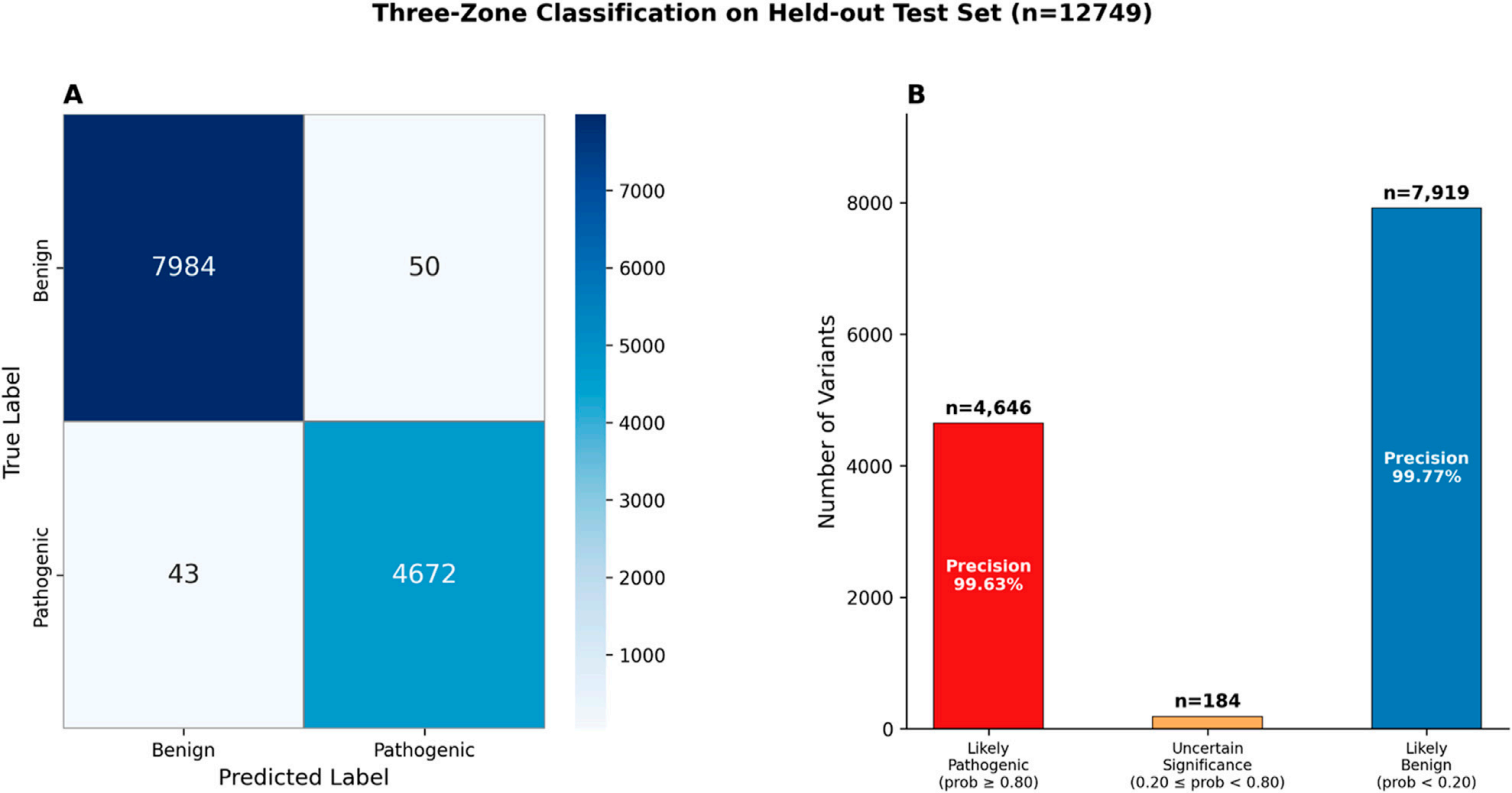
Three-zone classification of the held-out test set (n = 12,749). **(A)** Confusion matrix of the tuned Random Forest classifier. **(B)** Variant distribution across the three classification zones at adopted thresholds of ≥0.8 (Pathogenic/Likely Pathogenic) and ≤0.2 (Benign/Likely Benign) with precision reported for high-confidence zones.

At the adopted thresholds, the classifier achieved a pathogenic precision of 99.63% and a benign precision of 99.77% on the held-out test set, with 98.6% of the 12,749 test variants receiving a high-confidence classification. The predicted probability distribution across the test set showed a strongly bimodal pattern, with the majority of variants assigned high-confidence pathogenic or benign predictions and only a small proportion falling within the uncertain zone. Whereas the resulting three-zone classification scheme assigned variants with predicted probability at ≥ 0.80 as Pathogenic/Likely Pathogenic/, those ≤0.20 as Benign/Likely Benign and those falling between the two thresholds as VUS, reflecting genuine model uncertainty rather than a forced binary decision (Figure 5), the held-out test set of 12,749 variants yielded the significant outcomes (Table 4).

**TABLE 4:** Three-zone classification outcomes on the held-out test set (n = 12,749).

Classification	Total variants	Correctly classified
High confidence pathogenic (prob ≥ 0.80)	4,646	4,629/4,646 (99.63%)
High confidence benign (prob ≤ 0.20)	7,919	7,901/7,919 (99.77%**)**
Uncertain zone (0.20 ≤ prob <0.80)	184 variants	Retained as VUS (1.4%)

Misclassification analysis of the held-out test set revealed that the 24 false negative predictions (Pathogenic predicted as Benign) were enriched for intronic (29%), regulatory (25%) and splice region (21%) variants with Modifier or Moderate functional impact, suggesting that non-coding and regulatory pathogenic variants with low computational deleteriousness scores represent the primary challenge for the classifier. The 17 false positive predictions (Benign predicted as Pathogenic) exhibited high mean CADD Phred scores (mean = 27.5) and very low population frequencies (mean MAX_AF = 0.0004), representing variants that share computational signatures with pathogenic variants but are functionally neutral, a known challenge in variant classification referred to as Benign mimics.

### 3.4 SHAP-based model explainability

**FIGURE 6:**
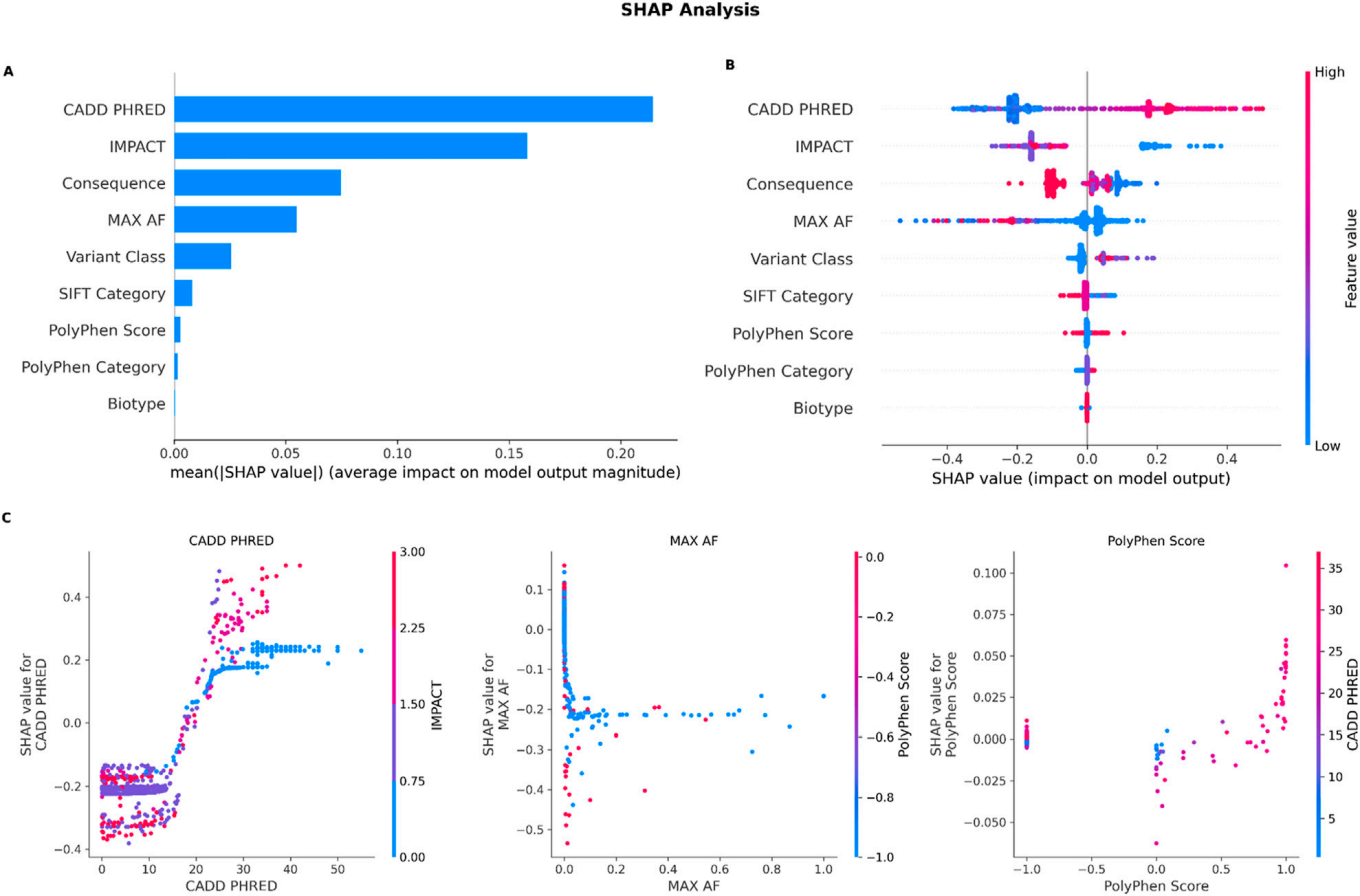
**(A)** Global feature importance of the tuned Random Forest classifier quantified by mean absolute SHAP values across 1,000 randomly sampled held-out test variants. **(B)** SHAP beeswarm plot illustrating the direction and magnitude of each feature’s contribution to predicted pathogenicity across 1,000 sampled held-out test variants. Each point represents one variant, coloured by feature value from low (blue) to high (red). Positive SHAP values indicate a push toward pathogenicity; negative values indicate a push toward benign classification. **(C)** SHAP dependence plots for the three most important features: genome-wide deleteriousness score, functional impact tier and missense pathogenicity score illustrating the relationship between individual feature values and their corresponding SHAP contributions to predicted pathogenicity across 1,000 sampled held-out test variants.

To assess the biological interpretability of the trained Random Forest classifier, SHapley Additive exPlanations values were computed on a random sample of 1,000 held-out test variants using the TreeExplainer algorithm. Global feature importance was quantified as the mean absolute SHAP value per feature across all sampled variants, providing a model-agnostic ranking of individual feature contributions to predicted pathogenicity (Table 5). The genome-wide deleteriousness score emerged as the strongest single predictor of pathogenicity (mean |SHAP| = 0.214) followed by the functional impact tier assigned by variant annotation (0.158), molecular consequence category (0.075) and maximum observed population allele frequency across global databases (0.055). Variant class, missense deleteriousness category, missense pathogenicity score and transcript biotype contributed progressively smaller effects, with transcript biotype contributing negligibly (mean |SHAP| = 0.0003), suggesting its potential removal in future model iterations without performance loss. The beeswarm plot confirmed the directionality of feature contributions across all sampled variants, with higher genome-wide deleteriousness scores and high-impact functional annotations consistently associated with positive SHAP values driving predictions toward pathogenicity and greater population allele frequency associated with negative SHAP values driving predictions toward benign classifications (Figure 6). Dependence plots for the three most contributory features further illustrated linear relationships between feature values and their SHAP contributions.

**TABLE 5:** Global feature importance of the tuned Random Forest classifier quantified by mean absolute SHAP values across 1,000 randomly sampled held-out test variants, ranked in descending order of contribution to predicted pathogenicity.

Rank	Feature	Mean |SHAP|	Biological interpretation
1	CADD phred score	0.214	Genome-wide deleteriousness
2	VEP IMPACT category	0.158	Functional impact tier
3	Variant consequence	0.075	Molecular consequence type
4	MAX population AF	0.055	Population rarity signal
5	Variant class	0.025	SNV/indel/deletion type
6	SIFT category	0.008	Missense deleteriousness
7	PolyPhen category	0.003	Missense pathogenicity
8	PolyPhen score	0.001	Missense category
9	Biotype	0.0003	Transcript type

### 3.5 VUS reclassification

**FIGURE 7:**
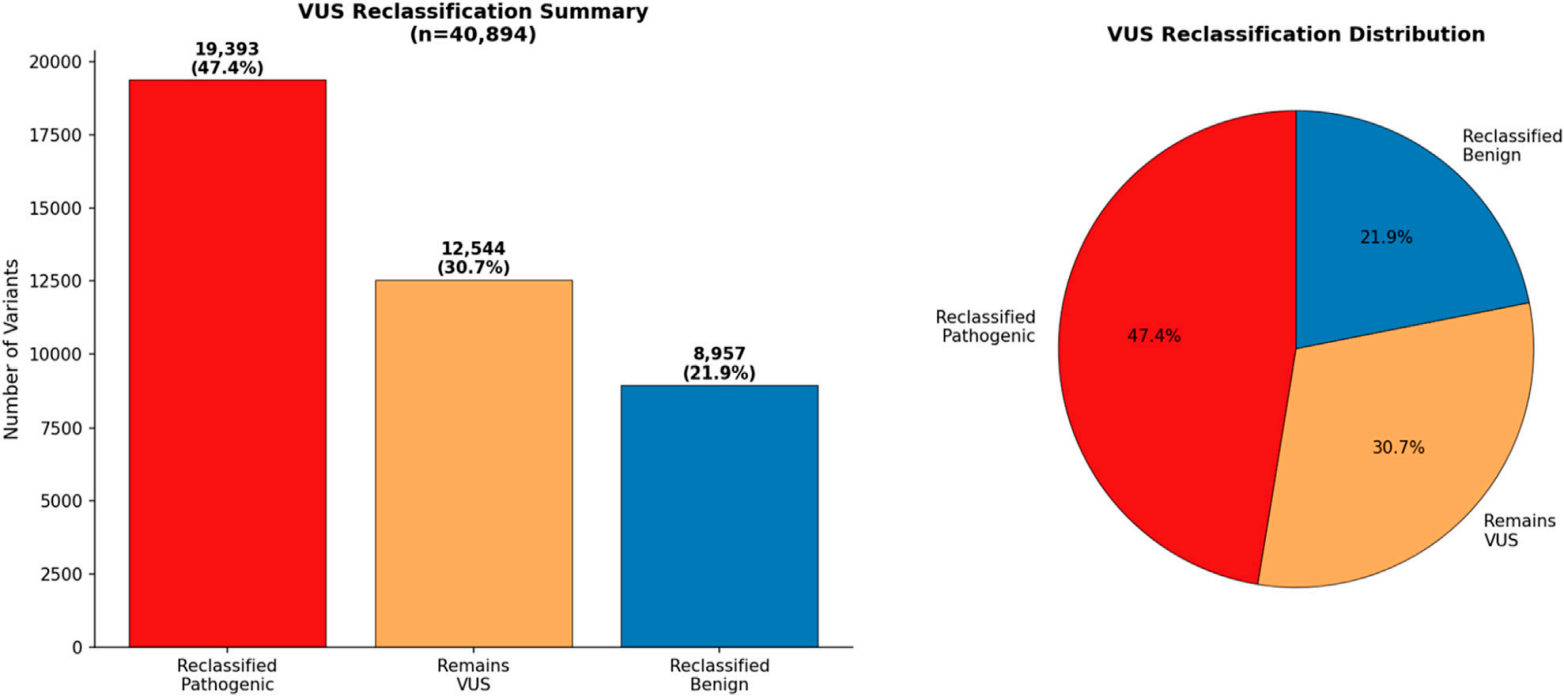
VUS reclassification outcomes across the full inference set (40,894) at adopted thresholds of ≥0.80 (Pathogenic) and ≤0.20 (Benign).

The Random Forest classifier was applied to the 40,894 VUS withheld from training the model. Of these, 19,393 (47.4%) were reclassified as Pathogenic/Likely pathogenic, 8,957 (21.9%) as Benign/Likely Benign and 12,544 (30.7%) were retained as VUS (Figure 7). The higher proportion of Likely Pathogenic reclassifications reflects the clinical ascertainment bias inherent to ClinVar VUS submissions, which are predominantly derived from patients undergoing hereditary cancer panel testing and therefore enriched for rare, damaging variants in disease-relevant genes. To assess sensitivity of reclassification outcomes to threshold selection, variant distributions were evaluated across Pathogenic probability thresholds of 0.70, 0.80, 0.90 and 0.95 (Table 6).

**TABLE 6:** Sensitivity analysis of VUS reclassification outcomes across Pathogenic probability thresholds applied to the VUS inference set (n = 40,894).

Pathogenic threshold	Reclassified pathogenic	Reclassified benign	Remains VUS
≥0.70	22,012 (53.8%)	8957 (21.9%)	9925 (24.3%)
**≥0.80**	**19393 (47.4%)**	**8957 (21.9%)**	**12544 (30.7%)**
≥0.90	14,807 (36.2%)	8957 (21.9%)	17,130 (41.9%)
≥0.95	11,201 (27.4%)	8957 (21.9%)	20736 (50.7%)

The Benign threshold was held fixed at 0.20 throughout. The adopted threshold of 0.80 is highlighted.

**FIGURE 8:**
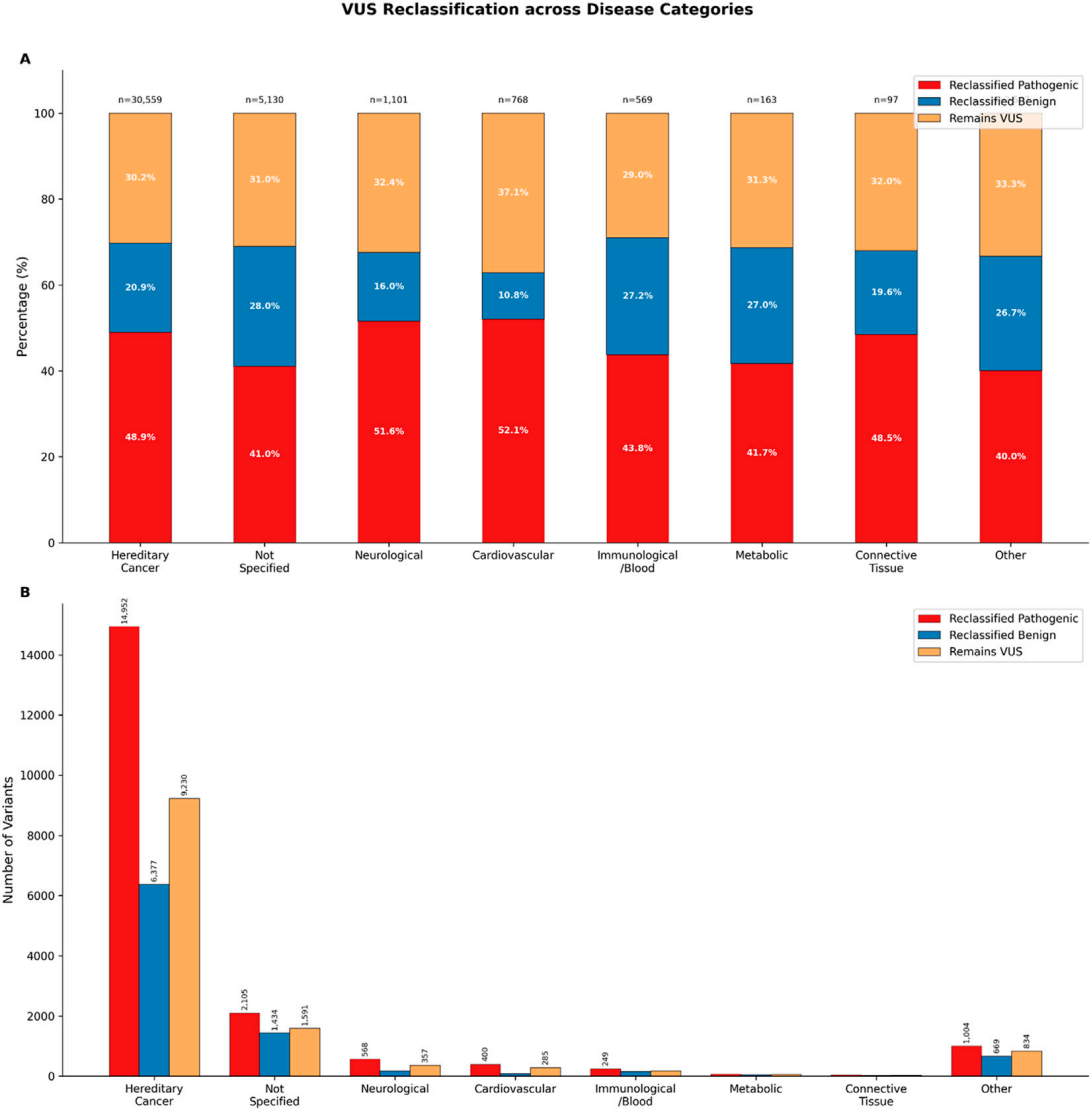
Distribution of VUS reclassification outcomes across disease categories. Hereditary cancer conditions account for 74.7% of all reclassified variants. **(A)** VUS reclassification by disease category(%) **(B)** VUS reclassification by disease category (Counts).

Of the 40,894 VUS, 74.7% (n = 30,559) were associated with hereditary cancer conditions as annotated in ClinVar. Among the 19,393 variants reclassified as Likely Pathogenic, 14,952 (77.1%) were associated with hereditary cancer syndromes including hereditary cancer-predisposing syndrome, hereditary nonpolyposis colorectal neoplasms, familial cancer of breast, familial adenomatous polyposis, Lynch syndrome, ataxia-telangiectasia and neurofibromatosis type 1, among others (Figure 8). Beyond hereditary cancer, reclassified variants additionally spanned neurological (n = 1,101), cardiovascular (n = 768), and immunological and blood disorders (n = 569), demonstrating broader applicability of the framework. Gene-level analysis revealed enrichment of Likely Pathogenic reclassifications in established hereditary cancer susceptibility genes including APC, ATM, MSH6, BRIP1, CHEK2, NF1, BRCA2, BARD1 and PMS2 (S2).

### 3.6 External validation accurately predicted ca. 89% of ENIGMA variants

Application of the tuned Random Forest classifier to the 7,446 annotated ENIGMA variants (16 variants excluded due to absence of CADD scores) yielded an overall concordance of 98.83% with ENIGMA expert classifications (Table 7; Supplementary Table S3). The classifier achieved a ROC-AUC of 1.0000 on the ENIGMA validation set, with 99.0% of variants receiving high-confidence classifications.

**TABLE 7:** External validation performance metrics on 7446 ENIGMA-classified BRCA1/BRCA2 variants from the BRCA Exchange database.

Metric	Value
Total variants	7,446
Overall concordance	98.83% (7,359/7,446)
High confidence coverage	99.0% (7,370/7,446)
ROC-AUC	1.000
Pathogenic precision	1.00
Pathogenic recall	1.00
Benign precision	1.00
Benign recall	1.00

**FIGURE 9:**
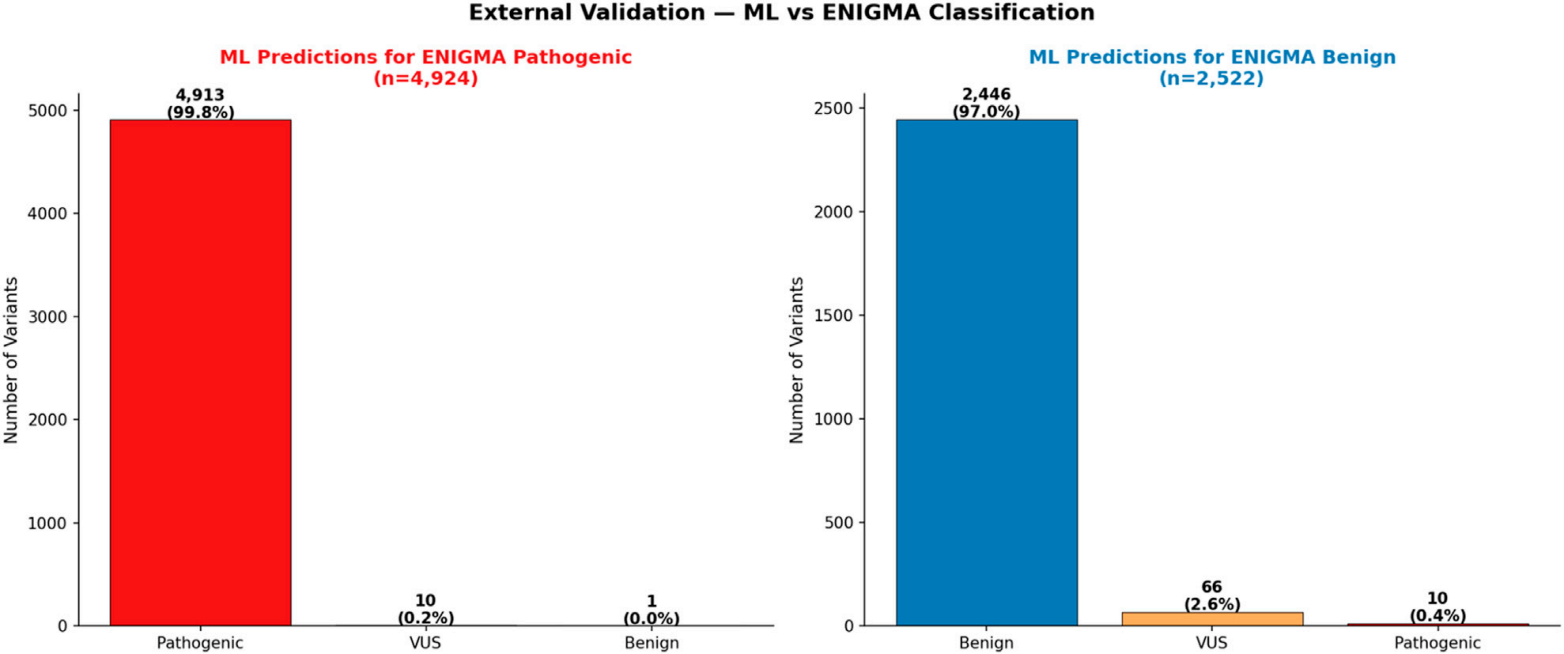
ML prediction distribution for ENIGMA Pathogenic (left) and Benign (right) variants, showing the proportion of correctly classified, incorrectly classified, and uncertain zone variants within each ENIGMA classification group.

Among the 4924 ENIGMA Pathogenic variants, 4,913 (99.8%) were correctly predicted as Pathogenic, 1 (0.02%) was incorrectly predicted as Benign and 10 (0.2%) fell within the uncertain zone. Among the 2522 ENIGMA Benign variants, 2,446 (97.0%) were correctly predicted as Benign, 10 (0.4%) were incorrectly predicted as Pathogenic and 66 (2.6%) fell within the uncertain zone. The high concordance across both classes on a completely independent dataset confirms the generalizability of the trained classifier and its applicability to hereditary cancer variants beyond the ClinVar training distribution (Figure 9).

To specifically validate the VUS reclassification performance, 671 variants classified as VUS in ClinVar but with definitive ENIGMA classifications were identified from the BRCA Exchange database. These variants represent cases where ClinVar lacked sufficient evidence for classification but ENIGMA independently assigned definitive classifications based on functional and segregation evidence, providing a ground truth for evaluating the accuracy of computational VUS reclassification.

**FIGURE 10:**
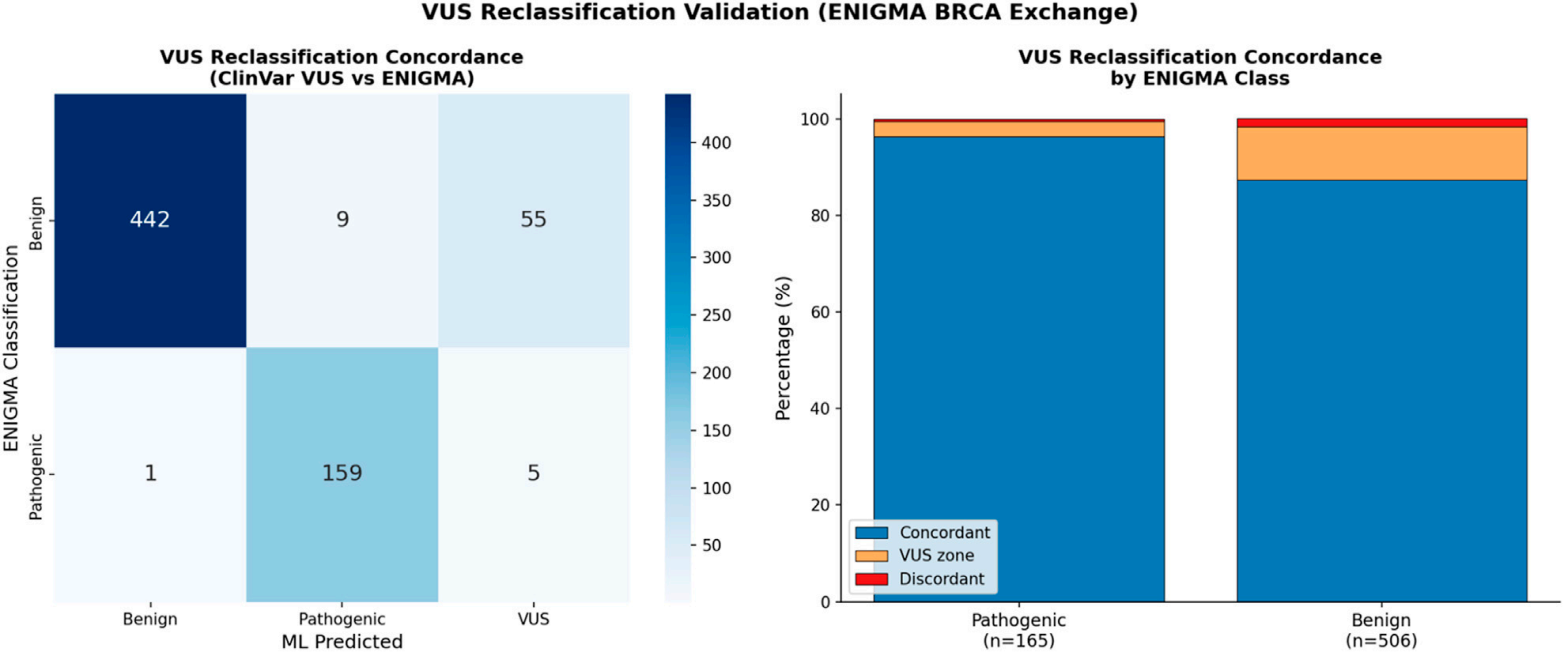
VUS reclassification concordance against ENIGMA expert classifications for 671 ClinVar VUS variants. The left panel shows the confusion matrix of ML predictions versus ENIGMA classifications; the right panel shows concordance rates for Pathogenic and Benign ENIGMA groups.

Among the 671 matched variants, 165 carried ENIGMA Pathogenic classifications and 506 carried ENIGMA Benign classifications. The tuned Random Forest classifier achieved an overall concordance of 89.57% (601/671) with ENIGMA classifications on this VUS validation set (Table 8; Figure 10). Pathogenic concordance was 96.4% (159/165), with only 1 variant incorrectly predicted as Benign and 5 retained in the uncertain zone. Benign concordance was 87.4% (442/506) with 9 variants incorrectly predicted as Pathogenic and 55 retained in the uncertain zone. The lower Benign concordance reflects the inherent challenge of computationally identifying functionally neutral variants that lack strong population frequency evidence, a known limitation of sequence based variant classification approaches and is consistent with the Benign mimic phenomenon observed in the held-out test set analysis.

**TABLE 8:** VUS reclassification concordance against ENIGMA expert classifications for 671 variants classified as VUS in ClinVar but definitively classified by ENIGMA.

Category	ENIGMA pathogenic (n = 165)	ENIGMA benign (n = 506)	Total (n = 671)
Correctly predicted	159 (96.4%)	442 (87.4%)	601 (89.57%)
Incorrectly predicted	1 (0.6%)	9 (1.8%)	10 (1.49%)
VUS	5 (3.0%)	55 (10.9%)	60 (8.94%)

This study presents a machine learning framework for the computational reclassification of VUS in hereditary cancer genomics, trained on 104,646 high-confidence ClinVar germline variants with strict variant-level data partitioning, systematic hyperparameter optimization and external validation on an independent expert-curated dataset.

The tuned Random Forest classifier achieved a test set ROC-AUC of 0.9995 (95% CI: 0.9993–0.9997) with stable 10-fold cross-validation performance (0.9992 ± 0.0004), demonstrating robust generalization to VUS. The narrow ROC-AUC range across all four evaluated classifiers (0.9957–0.9995) confirms that discriminative signal is primarily determined by the biological feature set rather than classifier-specific architecture, consistent with observations across the variant classification literature. SHAP-based explainability analysis confirmed that the top predictive features correspond directly to primary ACMG/AMP evidence criteria. CADD Phred score (mean |SHAP| = 0.214) aligns with PP3 (computational evidence/predictive pathogenicity), maximum population allele frequency (mean |SHAP| = 0.055) aligns with Pathogenic Moderate (PM) 2 (absent from population databases) and Benign Alone (BA) 1 (high allele frequency) and variant consequence class (mean |SHAP| = 0.075) aligns with Pathogenic Very Strong (PVS)1. We argue that this criteria provides biological validation addressing a potential barrier for a VUS to be misclassified, thereby avoiding the model to overfit with transcript-level splitting in VEP-annotated datasets.

Clinically grounded probability thresholds of ≥0.80 and ≤0.20 were adopted consistent with the Bayesian ACMG/AMP framework, achieving Pathogenic precision of 99.63% and Benign precision of 99.77% with 98.6% of test variants receiving high-confidence classifications. The three-zone classification scheme mirrors the ACMG/AMP system, with variants in the uncertain zone flagged for further investigation rather than forced into a binary decision. False negative predictions (n = 24) were enriched for intronic, regulatory and splice region variants with low computational deleteriousness scores, reflecting the likelihood of a non-coding variant to be classified. Incorporation of SpliceAI scores in future iterations may improve sensitivity for this variant class as false positive predictions (n = 17) represented significant functionally neutral variants further augmenting orthogonal functional validation (Jaganathan et al., 2019). Of 40,894 ClinVar VUS, 47.4% were reclassified as Pathogenic and 21.9% as Benign with 30.7% retained as VUS. The higher pathogenic proportion reflects clinical ascertainment bias in ClinVar VUS submissions, which are enriched for rare damaging variants from hereditary cancer panel testing, supported by the markedly higher mean CADD Phred score (26.16 vs. 12.07) and 40-fold lower population allele frequency of reclassified Pathogenic variants (Supplementary Table S1). Sensitivity analysis confirmed the reclassification distribution is threshold-dependent, with the adopted threshold of ≥0.80 representing a balanced operating point between sensitivity and clinical confidence. Of all VUS, 74.7% (n = 30,559) were associated with hereditary cancer conditions, with reclassified Pathogenic variants enriched in APC, ATM, MSH6, BRIP1 and CHEK2 genes with the highest VUS burden in hereditary cancer panels. The framework additionally demonstrated applicability across neurological, cardiovascular and immunological conditions, reflecting the disease-agnostic nature of the feature set. Computational reclassification represents a probabilistic prioritization framework rather than definitive clinical reclassification; reclassified variants should be considered high-priority candidates for functional investigation or expert panel review consistent with ACMG/AMP guidelines.

External validation on 7462 ENIGMA-classified *BRCA1/BRCA2* variants from the BRCA Exchange database demonstrated 98.83% overall concordance and ROC-AUC of 1.0000, confirming generalizability to an independent hereditary cancer dataset (Supplementary Table S2). VUS reclassification concordance against 671 ClinVar VUS with ENIGMA ground truth classifications achieved 89.57% overall agreement (Supplementary Table S3). The lower Benign concordance reflects the inherent challenge of computationally identifying functionally neutral variants that share computational signatures with pathogenic variants which is a known limitation of sequence-based variant classification approaches. A complete list of all genes and their associated variants included in the training dataset is provided in Supplementary Table S4. Furthermore, this list constitutes variants associated with the diseases and among them ca. 38% of them are VUS which is in agreement with the publicly available databases.

The performance of the current framework compares favourably with existing approaches (Table 9). Khandakji and Mifsud (2022) developed an XGBoost classifier trained exclusively on ENIGMA-curated BRCA2 variants, achieving 99.9% accuracy on held-out expert-reviewed variants and 91.3% on functional VUS, however, this approach is restricted to a single gene and does not incorporate clinically grounded thresholds or variant-level explainability. Kang et al. (2023) demonstrated 95%–98% accuracy for *BRCA1/BRCA2* missense variants using gene-specific Random Forest and XGBoost models across 28 hereditary cancer genes, but was limited to missense variants only and lacked external validation. The current framework extends these approaches in four important ways: first, training on 104,646 high-confidence variants spanning 3,159 genes and all variant consequence classes enables genome-wide applicability beyond gene specific or missense restricted models. Second, systematic hyperparameter optimization using grid search with five-fold cross validation was performed confirming that performance is near-optimal for the given feature set. Third, clinically grounded probability thresholds of ≥0.80 and ≤0.20 anchored to the Bayesian ACMG/AMP framework achieve Pathogenic and Benign precision exceeding 99%. Four, SHAP-based explainability provides individual variant-level reasoning chains directly linked to ACMG/AMP evidence criteria which is a transparency feature absent from both comparison studies and directly relevant to clinical adoption of ML-based variant classification tools. We further aim to incorporate SpliceAI scores, extension of external validation to broader hereditary cancer gene panels and prospective clinical validation in multi-center hereditary cancer cohorts to assess real-world utility in diagnostic genomics workflows.

**TABLE 9:** Comparison of the proposed framework with published machine learning approaches for hereditary cancer variant classification.

Feature	Khandakji and Mifsud (2022)	Kang et al. (2023)	Current study
Genes covered	BRCA2 only	28 hereditary cancer genes	3,159 genes
Variant types	All types	Missense only	All consequence types
Training size	ENIGMA-curated (small)	1,068 variants	104,646 variants (ClinVar)
Classifier	XGBoost	RF, XGBoost	LR, SVM, RF, XGBoost
Hyperparameter tuning	Not reported	Not reported	GridSearchCV 5-fold CV
External validation	No	No	Yes - 7462 ENIGMA variants (98.83% concordance)
SHAP explainability	No	No	Yes - SHAP (variant-level)
ACMG-anchored thresholds	No	No	Yes - ≥0.80/≤0.20 (bayesian ACMG/AMP)
Performance	99.9% accuracy (labeled); 91.3% (functional VUS)	95%–98% accuracy (BRCA1/2 missense)	99.95% accuracy (CI: 0.9993–0.9997); VUS concordance 89.57%

One of the key strengths of this study is the use of an independent external dataset for model validation. Although validation was conducted using the expert-curated ENIGMA BRCA Exchange dataset, which includes only *BRCA1* and *BRCA2* variants, these genes are among the most extensively studied hereditary cancer susceptibility genes and have highly reliable classifications established through standardized, evidence-based guidelines. As a result, the BRCA Exchange represents one of the most robust publicly available resources for benchmarking computational methods in discerning germline variant classification. Whereas external validation using expert-curated datasets is widely recognized as an appropriate approach for evaluating the generalizability of machine learning models, it has been adopted for developing variant pathogenicity predictors as described earlier (Table 9). The observed 89% concordance with ENIGMA classifications, therefore, provides independent evidence that the proposed model can accurately classify previously unseen variants. Importantly, unlike gene-specific prediction models, our classifier was trained on a comprehensive collection of ClinVar variants spanning 3,159 genes, allowing it to learn generalized relationships between variant features and clinical significance associated with gene-specific patterns. Nonetheless, evaluation on the independently curated BRCA Exchange dataset was intended to assess the model’s ability to generalize using a high-confidence reference standard demonstrating performance limited to BRCA-associated genes. However, we acknowledge that validation using additional expert-curated datasets representing a broader range of hereditary cancer genes would further strengthen evidence of the model’s generalizability and clinical applicability. Given that comparable large-scale, publicly available expert-reviewed datasets remain limited, our future work is aimed to focus on expanding external validation as more high-quality gene-specific curation resources and diverse clinical cohorts as they become available.

## 4 Conclusion

We present a transparent, reproducible and externally validated machine learning framework for VUS prioritization in hereditary cancer genomics. Taken together, our model was trained on 104,646 high-confidence ClinVar germline variants, wherein the tuned Random Forest classifier achieved 99.95% whereas with external validation resulted in concordance of 98.83% on 7462 ENIGMA-classified variants. Applied to 40,894 ClinVar VUS, 69.3% were computationally reclassified with SHAP analysis confirming predictions are driven by features concordant with ACMG/AMP evidence criteria. We hope the framework could possibly enhance our understanding with VUS reclassification thereby assisting clinical geneticists ascertain the variants with better interpretation bridging the gap of the diagnostic odyssey.

## Significance statement

Variants of uncertain significance (VUS) represent one of the most pressing challenges in clinical genomics; their unknown disease impact creates uncertainty for patients, clinicians, and genetic counselors alike. We developed a machine learning framework that trains exclusively on variants with established clinical significance and applies the learned decision boundary to reclassify VUS, avoiding the circular logic of treating uncertain labels as a training target. Using transcript-level variant effect predictor (VEP) annotations from ClinVar and a Random Forest classifier achieving an AUC-ROC of 0.961, we demonstrate that 36.9% of ClinVar VUS can be reclassified with greater than 99% precision using clinically-grounded probability thresholds aligned with ACMG/AMP guidelines. Applied to patient data, the framework identified two uncertain variants in cancer-relevant genes; MAP2K4 and FANCL, both rare, novel, and absent from ClinVar, representing candidates for functional follow-up. SHAP-based explainability confirms the model learns biologically meaningful signals rather than spurious correlations, offering a transparent and reproducible tool for variant triaging in hereditary cancer genomics.

## Data Availability

The original contributions presented in the study are included in the article/Supplementary Material, further inquiries can be directed to the corresponding author.
